# Evaluating The Effective Factors in Pregnancy after Intrauterine Insemination: A Retrospective Study

**DOI:** 10.22074/ijfs.2015.4544

**Published:** 2015-10-31

**Authors:** Firoozeh Ghaffari, Shahideh Jahanian Sadatmahalleh, Mohammad Reza Akhoond, Poopak Eftekhari Yazdi, Zahra Zolfaghari

**Affiliations:** 1 Department of Endocrinology and Female Infertility, Reproductive Biomedicine Research Center, Royan Institute for Reproductive Biomedicine, ACECR, Tehran, Iran; 2 Department of Midwifery and Reproductive Health, Faculty of Medical Sciences, Tarbiat Modares University, Tehran, Iran; 3 Department of Statistics, Mathematical Science and Computer Faculty, Shahid Chamran University, Ahwaz, Iran; 4 Department of Embryology, Reproductive Biomedicine Research Center, Royan Institute for Reproductive Biomedicine, ACECR, Tehran, Iran

**Keywords:** Pregnancy Rate, Infertility, Prognostic Factors

## Abstract

**Background:**

Controlled ovarian hyperstimulation (COH) in conjunction with intrauterine inseminations (IUI) are commonly used to treat infertile couples. In this study we evaluated the relationship between IUI outcome and special causes of infertility. We also aimed to examine parameters that might predict success following IUI.

**Materials and Methods:**

In this cross-sectional study, we included 994 IUI cycles in 803 couples who referred to the infertility Institute. All statistical analyses were performed by using SPSS program, t tests and chi-square. Stepwise multiple linear regression analysis was performed to compare the association between dependent and independent variables. Logistic regression was conducted to build a prediction model of the IUI outcome.

**Results:**

Overall pregnancy rate per completed cycle (16.5%) and live birth rate per cycle (14.5%). The mean age in the pregnant group was significantly lower than that of the non-pregnant group (P=0.01).There was an association between cause of infertility and clinical pregnancies (P<0.001). Logistic regression identified four significant factors in determining the success of the IUI [menstrual irregularites (OR:2.3, CI:1.6-3.4, P<0.001), duration of infertility (OR:0.8, CI:0.8-0.9, P<0.001), total dose of gonadotropin (OR:1.02, CI:1.003-1.04, P=0.02) and semen volume (OR:1.1, CI:1.008-1.2, P=0.03)] which were the most predictive of IUI success.

**Conclusion:**

Our study defined prognostic factors for pregnancy in COH+IUI. These variables can be integrated into a mathematical model to predict the chance of pregnancy rate in subsequent COH+IUI cycles.

## Introduction

Controlled ovarian hyperstimulation (COH) in conjunction with intrauterine inseminations (IUI) are commonly used to treat infertile couples (1). The most important indications for IUI are male subfertility, unexplained infertility, ovulatory dysfunction and cervical factor infertility (2). Several prognostic factors that determine IUI treatment outcome have been identified and include the woman’s age, duration of infertility, follicle number, endometrial thickness, numbers of sperm inseminated, sperm morphology, progressive motile sperm count, and cause of infertility (3-5). Tomlinson et al. (6) found no differences in age, duration of infertility, number of follicles, body mass index (BMI) and sperm quality in the pregnancy rates of patients undergoing IUI. Although IUI with or without ovarian stimulation is widely used, its success rate is lower than that of the assisted reproductive technologies (ART) (7). Nevertheless, in comparison to ART controlled ovarian hyperstimulation combined with intrauterine (COH+IUI) requires less frequent clinic visits, and is simple, relatively less invasive and comparatively inexpensive (8). Regardless of the method of assisted conception utilized, the couples always desire to know their chances of success. Thus, identifying the factors which are influential in the success rate is highly crucial. The purpose of this study was to identify the parameters which were most influential in the success rate using COH+IUI treatment modality. Using logistic regression, we were able to devise a mathematical model to predict the success rate in COH+IUI. The data presented, will enable the healthcare providers to counsel their patients about their chances of getting pregnant by using COH+IUI.

## Materials and Methods

In this cross-sectional study, we included 994 IUI cycles in 803 couples who referred to the infertility Institute between 2010-2012. This study was approved by the Institutional Review Board of the Royan Institute Research Center and the Royan Ethics Committee according to the Helsinki Declaration, signed informed written consent was obtained from all participants. All couples had attempted to conceive for at least one year prior to undergoing COH+IUIs. The women completed the self-administered questionnaire which was used to collect data about demographic, menstrual and obstetrical characteristics. A menstrual interval shorter than 21 days and longer than 35 days is defined as menstrual irregularities. Amount of bleeding is varied (9).

The study population comprised of all couples who were candidates for COH+IUI and had diagnoses of subfertile male infertility, polycystic ovary syndrome (PCOS), mild or minimal endometriosis or unexplained infertility and various ovulatory disorders. Ovulatory disorders included diminished ovarian reserve, PCOS and hypothalamic amenorrhea. Subfertile male infertility was defined as per criteria outlined by Molinaro et al. (10).

The following evaluations were performed prior to the initiation of COH+IUI. The women underwent cycle day 3 hormone evaluation [folliclestimulating hormone (FSH), luteinizing hormone (LH), estradiol (E_2_), thyroid-stimulating hormone (TSH) and prolactin] and assessment of tubal patency by using hysterosalpingogram (HSG) and/ or laparoscopy. Tubal patency of at least one tube was mandatory. In case of either a tubal abnormality in HSG or dysmenorrhea and dyspareunia, a laparoscopy was performed.

Inclusion criteria were: male factor, combined causes, Ovulatory disorder (Pco, diminished ovarian reserve, and hypothalamic amenorrhea), unexplained disorder, and all patients with normal TSH and prolactin levels. The couples with testicular atrophy, hydrosalpinx, anatomical abnormalities, infection, uterine fibroids, and systemic disease were excluded from participation.

All IUI cycles were performed with ovarian stimulation and included either clomiphene citrate (Iran Hormone Pharmaceutical Company, Iran), gonadotropin only, or letrozol (Femara, Novartis Pharma AG, Switzerland) or the combination of either clomiphene citrate or letrozol with gonadotropin. On days 11-12 of the menstrual cycle, we assessed follicular development and endometrial thickness by transvaginal ultrasound. If the endometrial thickness was <7 mm, 4 mg/per day E_2_ was administered (2 mg, Aburaihan Co., Iran) and continued during the luteal phase. Once a leading follicle of ≥18 mm was identified, human chorionic gonadotropin (5,000 IU IM, Pregnyl^®^, Darou Pakhsh Pharmaceutical, Iran) was administored to induce the final stage of oocyte maturation and a single IUI was planned 36-38 hours later. If more than five follicles ≥18 mm in size developed, the cycle was cancelled.

### Sperm preparation

Semen samples were obtained from patients who attended the unit for infertility treatments. Semen samples (n=994) were collected following 3-7 days of sexual abstinence. They were allowed to liquefy at (add room temperature or 37˚C) for 15-30 minutes and each was subject to an analysis according to the 2010 World health Organization (WHO) guidelines (11). After analysis, samples were prepared for IUI using to discontinuous density gradient centrifugation (DGC). For this purpose, we prepared a two layer gradient consisted of solutions of 100 and 50% Allgrade^®^ (LifeGobal, Belgium). The 50% concentration was made by diluting 100% Allgrade^®^ with Ham’s F10 (Sigma, USA) medium. The density gradients were performed by layering 2 mL of each concentration into a conical tube (15 mL, Falcon, Becton Dickinson, NJ, USA). These tubes were pre-incubated for at least 2 hours in a 37˚C incubator. After semen liquefaction, 2 mL of ejaculation was layered on the top of the Allgrade^®^ gradient and centrifuged for 30 minutes at 300 x g. After centrifugation, the sperm was collected at the bottom of the tube by a clean Pasteur pipette and transferred to a 5 mL clean tube (Falcon), and washed twice with Ham’s F10 medium by using centrifugation at 300 x g for 5 minutes. The pellet was resuspended in 1 mL of Ham’s F10 medium, then the sperm concentration and motility were evaluated. In our study the sperm analysis data haven’t been recorded after processing and sperm analysis data before processing was available.

Intrauterine inseminations was performed by a soft catheter (INDOVASIVE, Biorad, India) with an insemination volume of 0.6 mL. The IUI catheter was passed gently through the cervical canal until the tip passed the internal os. Then, the sperm suspension was deposited slowly through the uterine cavity. All patients were provided with luteal support by using cyclogest according to the treatment physician’s preference. Clinical pregnancy was defined as a positive pregnancy test followed by the presence of a gestational sac visualized by transvaginal sonography 4weeks after IUI.

In order to build a prediction model, we used stepwise logistic regression analysis, in which a P value of 0.15 was used as an entry criterion, whereas a P value of 0.10 was the threshold for a variable to stay in the model. We check the performance of the model by the area under the receiver operating characteristic (ROC) curve (AUC). An AUC of 0.5 indicates no discriminative performance, whereas an AUC of 1.0 indicates perfect discrimination.

Calibration of the model was assessed by comparing the predicted probability of pregnancy in a category of patients and the observed percentage of pregnant woman in that category. We first categorized the predicted probabilities of pregnancy in 10 groups, then we compared the mean predicted probability of pregnancy in that particular category with the observed probability, i.e. pregnancy rate in that category.

### Statistical analysis

All statistical analyses were done by using SPSS software (version 20, USA). Chi-square and t tests were used for analyses. We performed univariate logistic regression for each factor and reported the odds ratio (OR) and 95% confidence interval (CI). In order to predict the IUI result, we used multiple logistic regression analyses. Data were expressed as mean ± standard deviation (SD). A Pvalue of <0.05 was considered to be statistically significant.

## Results

We studied a total of 994 IUI cycles in 803 couples. Each couple underwent 1.23 ± 0.4 (mean ± SD) COH+IUI cycles (range: 1-3). Causes for infertility were: unexplained disorder (290, 29.2%), male factor (395, 39.7%), combined causes (108, 10.9%), and ovulatory disorder (201, 20.2%). In our study population, ovulatory disorders included diminished ovarian reserve, 0.5% (n=1), PCOS, 93.5% (n=188) and hypothalamic amenorrhea, 6% (n=12).

In our study combined cause including; ovulatory disorder and male factor 83.3% (n=90), tub peritoneal and male factor 6.5% (n=7), uterine factor and ovulatory disorder 3.7% (n=4), uterine factor and male factor 2.8% (n=3), male factor and recurrent abortion 1.9% (n=2), ovulatory disorder and recurrent abortion 0.9% (n=1), uterine factor & recurrent abortion 0.9% (n=1).

Table 1 compares the demographic characteristics between pregnant and nonpregnant women. The pregnancy rate in younger women was significantly higher than those of older women. In addition, an infertility duration of ≤4years was associated with a significantly higher pregnancy rate (OR:1.5, CI:1.1-2.2, P=0.01). Infertility type (primary or secondary) did not significantly affect the outcome. With regards to the diagnosis of infertility, the highest pregnancy rate (27.8%) was achieved in couples with combined infertility, whereas the lowest (13.4%) rate was observed in couples who suffered from male factor infertility (P<0.001, Table 1).

**Table 1:** Characteristics of study patients who underwent IUI


	Pregnant	Nonpregnant	OR (95% CI)	P value

Female age (Y) ^†*^	27.80 ± 3.69	28.62 ± 3.94	0.97 (0.90-0.98)	0.01
**Male age (Y) ^†^**	32.95 ± 4.57	32.41 ± 4.41	0.97 (0.93-1.01)	0.16
Menstrual irregularities^*^				
** No**	93 (13.5)	596 (86.5)	0.48 (0.34-0.67)	<0.001
** Yes**	72 (24.5)	222 (75.5)	1^§^	
**BMI (kg/m^2^)**	25.23 ± 4.32	25.41 ± 14.36	0.99 (0.98-1.01)	0.88
**Type of infertility- n (%)**				
** Primary**	142 (16.6)	708 (83.4)	1^§^	
** Secondary**	24 (16.7)	120 (83.3)	1.00 (0.62-1.61)	1.00
Duration of infertility (Y)^ *^	3.65 ± 2.4	4.38±2.8	0.89 (0.83-0.96)	0.002
Etiology of infertility- n (%)^*^				
** Male factor**	53 (13.4)	342(86.6)	1^§^	<0.001
** Unexplained disorder**	40 (13.8)	250(86.2)	1.03 (0.66-1.60)	
** Ovulatory disorder**	42 (20.9)	159(79.1)	1.70 (1.09-2.66)	
** Combined**	30 (27.8)	78(72.2)	2.48 (1.48-4.13)	


IUI; Intrauterine insemination, OR; Odds ratio, CI; Confidence interval, BMI; Body mass index,†; Values are mean ± SD, §; Reference category and *; P<0.05 was considered as statistically significant.

The pregnancy rates according to female characteristics and sperm parameters (according to Strict Criteria) are summarized in table 2. Pregnancy rate was not related to sperm count. There were no significant differences in total sperm concentration among the pregnant and nonpregnant study population. Sperm parameters did not significantly affect the outcome of COH+IUI treatment. Seminal volume did not significantly affect the success of COH+IUI. The total dose of gonadotropin in nonpregnant women was significantly lower than that of the pregnant women (P=0.03, Table 2).

No significant difference was found between the two groups in different types of gonadotropins (data not shown).

Pregnancy outcome, in our study included, there were 3 (1.8%) ectopic pregnancies, 9 spontaneous miscarriages of which 5 (3%) occurred during the first trimester and 4 (2.4%) during the second trimester; 8 (4.8%) cases of blighted ovum, and 145 (87.9%) live births. This corresponded to an ongoing pregnancy rate of 14.9% (149/994) per IUI cycle. Of the 165 clinical pregnancies (ongoing pregnancies and early pregnancy loss), 22 were twin pregnancies (13.3%). There were 7 (4.2%) triplet pregnancies, of which one ended with a late abortion and another terminated at 24 weeks from which no fetus survived. Of the remaining triplet pregnancies, two mothers gave birth at 32 and 34 weeks (two healthy sets of one girl and two boys) and 3 triplet pregnancies were reduced to twins. The mean birth weight was 1488 ± 395 g and mean gestational age at delivery was estimated to be 32 weeks for the triplet pregnancies that reduced to twins. Of these, all neonates were well and healthy. The mean birth weight of singletons was 3000 ± 525.7 g, twins weighed 2081 ± 557.4 g and triplets weighed 1588.3 ± 549.1 g. All singletons ended at 28-40 weeks and twins at 30-38 weeks.

We used linear-by-linear test for calculation, the correlation between age of women and clinical, ongoing and multiple pregnancies rate. When one of the variables is ordinal and the other variable is ordinal or nominal with 2 level, this trend test can be used (12).

The proportion of clinical, ongoing and multiple pregnancies, decreased with age (P=0.041, P=0.044 and P=0.046, respectively, Table 3).

**Table 2:** Cycle parameters of the patients who underwent IUI


	Pregnant	Nonpregnant	OR (95% CI)	P value

Total dose of gonadotropin ^†*^	589.21 ± 554.61	508.43 ± 420.62	1.00 (1.00±1.0001)	0.03
**Serum FSH level on day 3 (IU/ml) ^†^**	6.36 ± 3.91	6.38 ± 2.50	0.99 (0.94-1.05)	0.93
**Serum LH level on day 3 (IU/ml)^ †^**	5.84 ± 3.52	5.94 ± 3.73	0.99 (0.94-1.03)	0.75
**Serum estradial level on day 3 (Pg/ml)^ †^**	51.78 ± 40.66	63.98 ± 106.69	0.99 (0.99-1.007)	0.72
**Sperm count (× 10^6^/ml)**	53.10 ± 25.32	51.71 ± 28.92	1.002 (0.99-1.008)	0.55
**Total sperm count**	175.93 ± 100.57	161.07 ± 107.43	1.001 (0.99-1.003)	0.10
**Total motile sperm (×10^6^/ml)**				
**<10**	1 (14.3)	6 (85.7)	0.81 (0.09-6.78)	
**10-20**	6 (9.5)	57 (90.5)	0.51 (0.21-1.20)	
**>20**	158 (17)	766 (83)	1^§^	0.29
**Normal morphology– n (%)**				
**≤10**	116 (16.5)	589 (83.5)	1^§^	0.89
**>10**	48 (16.8)	238 (83.2)	1.02 (0.70-1.48)	
**Semen volume**	3.59 ± 1.82	3.31 ± 1.64	1.09 (0.99-1.20)	0.054
**Endometrial thickness (mm) - n (%)**				
**<6**	3 (25)	9 (75)	1^§^	0.40
**≥6**	162 (16.5)	820 (83.5)	0.57 (0.15-2.13)	


IUI; Intrauterine insemination, OR; Odds ratio, CI; Confidence interval, FSH; Follicle-stimulating hormone , LH; Luteinizing hormone , †; Values are mean ± SD, §; Reference category,*; Significant statistical differences between the two groups.

**Table 3:** Clinical and ongoing pregnancy rates per couple and the frequency of multiple pregnancies for women according to age group


Age (Y)	Clinical pregnancy/ couple % (n)^*^	Ongoing pregnancy/ couple % (n)^*^	Multiple pregnancy/clinical pregnancy % (n)^*^

≤30	22.2 (124/558)	20.1 (112/558)	20.1 (25/124)
31-35	18 (37/206)	16.5 (34/206)	10.8 (4/37)
36-40	10.3 (4/39)	7.7 (3/39)	0
Total	20.5 (165/803)	18.6 (149/803)	17.5 (29/165)
P value	0.041	0.044	0.046


*; Significant statistical differences between the groups.

Of the 145 (87.8%) live births, 139 resulted in live deliveries at term, 123 (88.5%) patients underwent caesarean sections and 16 (11.5%) had normal vaginal deliveries. There were no major congenital anomalies reported. The live birth rate/ cycle was 14.5% (145/994).

The clinical pregnancy rate per couple was 20.5% (165/803) with an ongoing pregnancy rate per couple of 18.5% (149/803). Pregnancy rates per cycle were as follows: first (21%), second (19.4%) and third (15.3%).

Stepwise multiple linear regression analysis was performed to compare the association between dependent (total dose of gonadotropin) and independent (age, BMI, menstrual irregularities, duration of infertility, type of infertility, endometrial thickness, number of dominant follicle, etiology of infertility) variables. Age (P<0.001), menstrual irregularities (P<0.001), and duration of infertility (P=0.01) were the main variables that significantly influenced the total dose of gonadotropin in couples undergoing IUI (Table 4).

**Table 4:** Variables influencing total dose of gonadotropin in couples undergoing IUI


Variable	Coefficient	SE	P value^*^

**Age**	26.16	3.44	0.001
**Menstrual irregularities**	-118.36	29.56	0.001
**Duration of infertility**	10.97	4.94	0.02


*; P value multiple regression, IUI; Intrauterine insemination and SE; Standard error.

According to logistic regression, female age, duration of infertility, menstrual irregularities, seminal volume and total dose of gonadotropin were significantly associated with pregnancy outcome. Higher female age, prolonged duration of infertility and regular menstruation showed a negative association with pregnancy outcome, while seminal volume and total dose of gonadotropin were positively associated with pregnancy outcome (Table 5).

**Table 5:** Result of logistic regression analysis


Variable	OR	95% CI	P value

Duration of infertility (Y)^*^	0.86	0.80-0.93	<0.001
Menstrual irregularities *			
** No**	1^§^	1^§^	
** Yes**	2.37	1.65-3.40	<0.001
Semen volume*	1.11	1.008-1.22	0.03
Total dose of gonadotropin^*^	1.02	1.003-1.04	0.02
**Constant**	0.11		<0.001
AUC:0.65 (95% CI: 0.60-0.70)			


OR; Odds ratio, CI; Confidence interval, §; Reference category, *; Significant statistical differences and AUC; Area under curve.

**Fig.1:**
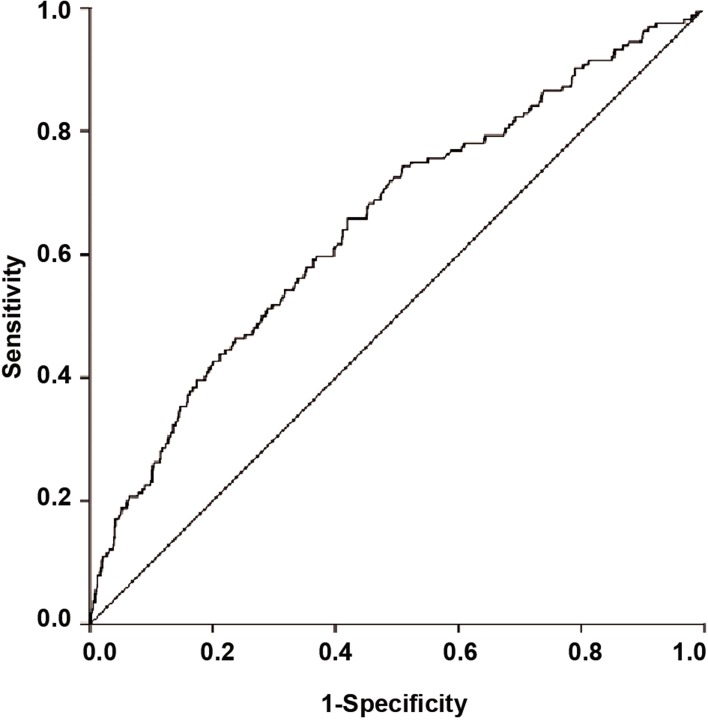
ROC curve for assessment of logistic regression discrimimative preformance. ROC; Receiver operating characteristic.

The ROC curve was used to assess the discriminative performance of the fitted logistic model (Fig .1). An AUC equal to 0.5 indicates no discriminative power whereas an AUC of 1.0 shows a perfect discrimination. In our study, the AUC for the fitted logistic model was found to be 0.65 with the 95% CI of 0.60 - 0.70, indicative of a reasonable prognostic potency for predicting pregnancy following COH+IUI.

With the data obtained, we were able to construct a formula for calculation of the probability of pregnancy following COH+IUI as carried out in our study (see below).

**Fig.2:**
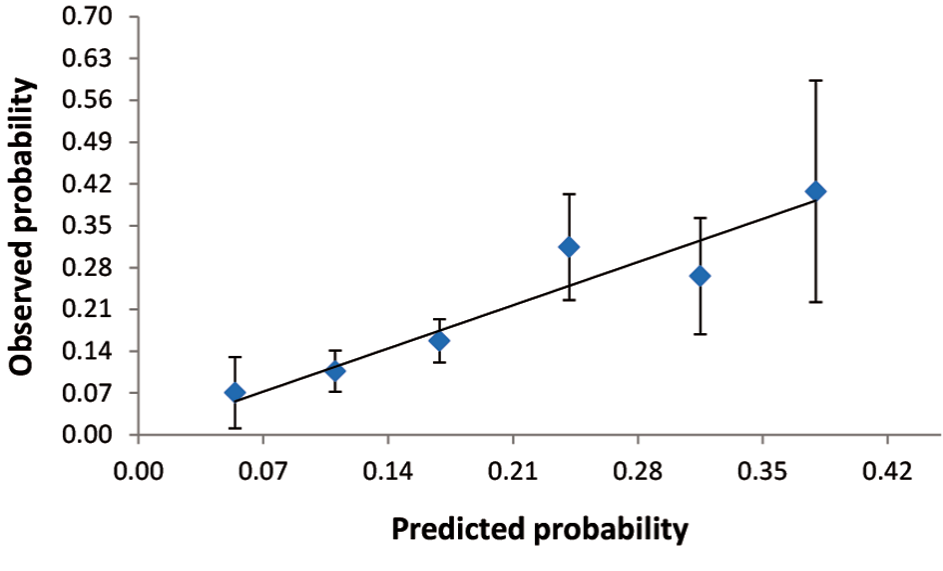
Calibration plot, showing the relationship between predicted and observed rate of pregnancy after intrauterine insemination (IUI).

In this formula, the duration of infertility is the number year each couple has been attempting to conceive without success. The menstrual history is=1 if history of menstrual is irregular and 0 if history of menstrual is regular. The performance of the prediction model for pregnancy following COH+IUI was calibrated as shown in figure 2. The predictive performance appears to be acceptable because the 95% confidence intervals of the observed pregnancy rates overlap with the predicted pregnancy rate.

$\mathrm{Probability of pregnancy after IUI}=\frac{e^{\left(-2.154-0.147\times \mathrm{Durationofinfertility}+0.865\times \mathrm{Menstrualhistory}+0.105\times \mathrm{Volume}+0.022\times \sqrt{\left(\mathrm{Total dose of gonadotropin}\right)}\right)}}{1+e^{\left(-2.154-0.147\times \mathrm{Durationofinfertility}+0.865\times \mathrm{Menstrualhistory}+0.105\times \mathrm{Volume}+0.022\times \sqrt{\left(\mathrm{Total dose of gonadotropin}\right)}\right)}}$

## Discussion

Among the various parameters that were studied, female age, duration of infertility, menstrual irregularities, semen volume, cause of infertility and the dose of gonadotropin significantly affected treatment success.

In the current study, we have shown a statistically significant association between reduced COH+IUI success rate and increased age. Several studies have illustrated the decline in pregnancy with advancing age, (13-15); however, Erdem et al. (15) did not find female age to be a prognostic factor in the prediction of a live birth in ovarian stimulation and IUI cycles. The current study, IUI was offered for women over the age of 40 years. According to studies, woman over the age of 40 are not good candidates for IUI (16, 17).

We observed a significant decrease in pregnancy rate with increased duration of infertility (OR: 0.8, CI: 0.8-0.9, P<0.001). This result was also supported by the results observed in a study by Kamath et al. (18). In another study (17), a significantly higher pregnancy rate (14.2%) was observed in couples with the duration of infertility of less than 6 years compared to 6.1% rate for those with the duration of more than 6 years. Merviel et al. (14) did not observe this difference. However, our findings indicate that the duration of infertility must be considered when counselted patients on their chances of a successful pregnancy.

Infertility type (primary or secondary) did not significantly affect the outcome of COH+IUI the result of which has been shown in some studies (14, 15). Our study found a significant effect of the total gonadotropin dose on outcome in pregnancies conceived by COH+IUI (P=0.03). The data were also evaluated to determine the variables which may influence the total dose of gonadotropins. It appears that women with higher age, those with menstrual irregularities and low number of dominant follicles as well as those with shorter duration of infertility should not be given high doses of gonadotropins. I argue with such a strong statement based on the findings presented in table 4. The data presented are simple correlation data. The only time you can make such a statement is when for example you give the same doses of gonadotropins to women of younger and older age and assess the outcome.

A total of 17.5% of the recorded clinical pregnancies after COH+IUI at our center were multiple pregnancies; no case of hyperstimulation was documented during the study period. Other studies have reported an incidence of twins (20%) and higher-order (39%) multiple pregnancies that were the result of ovulation induction (19, 20). Thus, centers should choose appropriate stimulation protocols and attempt to achieve a balance between the search for advanced success rate and suitable multiple pregnancy rates. The present study, no significant difference was found in endometrial thickness between pregnant women and those who did not become pregnant. This finding is similar to the result of Kamath et al. (18).

The information available at present study indicates that COH+IUI can be considered prior to more expensive IVF in patients that have combined (27.8% per cycle) and ovulatory disorder (20.9% per cycle) infertilities. The success rate was higher for ovulatory cases and for those who suffered from more than one etiological factor. The patients of this group have been diagnosed as the combination of mild male infertility and PCOS. Compared result has been reported regarding the highest success rate in an ovulatory patient (13, 15, 17). The clinical pregnancy rate was significantly higher in patients who had irregular menstruation. All of these patients were diagnosed with PCOS, according to the cause of infertility as discussed, the success rate was higher in an ovulatory patient. When the effect of the infertility etiology was assessed, there was a significantly lower pregnancy rate observed in endometriosis patients compared with women who had unexplained infertility (16). Peterson et al. (21) have found the average pregnancy rate for unexplained infertility to be 18%. Our result showed a 13.8% average pregnancy rate for unexplained infertility; however, Basirat and Esmaeilzadeh (22) determined that the etiology of infertility was not significantly different between pregnant and nonpregnant women (P=0.63).

Predictive sperm parameters for successful IUI have been controversial (23, 24). Total motile count (TMC) is a potential predictive factor for a successful COH+IUI (15). The pregnancy rates according to Kamath et al. (18) were as follows: a significantly higher pregnancy rate (18.2%) was observed when TMC was in the range of 10-20 million. TMC at a range of 5-10 million resulted in a 5.6% pregnancy rate, whereas in cases where TMC was <5 million, the rate was 2.7%. A TMC of <1 million was associated with poor pregnancy rates. When the TMC was <5 million, sperm morphology appeared to play an important role. A pregnancy rate of 18.4% was observed with a normal morphology compared to a rate of <5.4% with <30% morphology (18). In our study, although the sperm analysis data was not recorded after processing, the data prior to sperm processing was available. Sperm parameters did not significantly affect the success of COH+IUI. These results also confirm the findings achieved by other researchers (16, 25). In contrast, other studies have described several semen parameters that correlated with IUI outcome, such as the number of motile sperm (24, 26) and normal morphology (24). In the current study there was a lower pregnancy rate when the TMC was in the range of 10-20 million (9.5%). The pregnancy rate was 17% when the TMC was >20 million. The pregnancy rate increased when there was higher normal morphology (OR=1.02, 95% CI:0.7-1.4, Table 2). Generally, published studies have been inconsistent related to the association between the morphology readings and the success in IUI (21, 22).

We observed four parameters that significantly affected success: duration of infertility, menstrual irregularities, seminal volume and total dose of gonadotropin. In a previous study there were four prognostic factors: etiology and duration of infertility, number of treatment cycles, and number of pre ovulatory follicles (16). Kamath et al. (18). found a significant effect of the duration of infertility and TMC on outcome of pregnancies conceived by IUI.

Our study was a retrospective, no documents were available about the number of follicles and there was a limitation to our study. On the other hand, we did not have information about semen quality after processing and it was another ristriction.

## Conclusion

With the data obtained, we were able to construct a formula for to calculate the probability of pregnancy following COH+IUI as carried out in the present study. These results suggested that female age, duration of infertility, cause of infertility, menstrual irregularities, ejaculatory volume and total dose of gonadotropin be the most important prognostic factors in predicting successful outcome of IUI. A larger study population might assist with the formulation of a better predictive model for IUI success. Such information could be used by couples and clinicians during counseling participants to arrive at a decision with regards to their treatment options.
